# Associations between single nucleotide polymorphisms in the *FAS* pathway and acute kidney injury

**DOI:** 10.1186/s13054-015-1084-5

**Published:** 2015-10-19

**Authors:** Pavan Bhatraju, Christine Hsu, Paramita Mukherjee, Bradford J. Glavan, Amber Burt, Carmen Mikacenic, Jonathan Himmelfarb, Mark Wurfel

**Affiliations:** Pulmonary Critical Care Medicine, University of Washington, Harborview Medical Center, 325 Ninth Avenue, Box 359640, Seattle, WA 98104 USA; Kidney Research Institute, Division of Nephrology, University of Washington, Seattle, WA USA; Pulmonary Critical Care and Sleep Medicine, The Oregon Clinic, Portland, OR USA; Biostatistics University of Washington, Seattle, WA USA; Pulmonary and Critical Care, University of Washington, Seattle, WA USA

## Abstract

**Introduction:**

To determine whether single nucleotide polymorphisms (SNPs) in *FAS* and related genes are associated with acute kidney injury (AKI) in patients with acute respiratory distress syndrome (ARDS).

**Methods:**

We studied 401 (Caucasian N = 310 and African-American N = 91) patients aged ≥ 13 years with ALI who enrolled in the Fluid and Catheter Treatment Trial (FACTT) between 2000 and 2005 from 20 North American centers. We genotyped 367 SNPs in 45 genes of the Fas/Fas ligand pathway to identify associations between SNPs in Fas pathway genes and the development of AKI by day 2 after enrollment in FACTT, adapting Acute Kidney Injury Network (AKIN) criteria. Written informed consent was obtained from participants or legally authorized surrogates in the original FACTT study and available to use for secondary analysis.

**Results:**

In Caucasian patients, we identified associations between two SNPs and the incidence of AKI (stage 1 and above): rs1050851 and rs2233417; both are found within the gene for nuclear factor of kappa light polypeptide gene enhancer in B-cells inhibitor, alpha (*NFKBIA*). For rs1050851 and rs2233417, the odds ratios (ORs) were 2.34 (95 % confidence interval (CI) = 1.58–3.46, p = 1.06 × 10^−5^, FDR = 0.003) and 2.46 (CI = 1.61–3.76, p = 1.81 × 10^−5^, FDR = 0.003) for each minor allele, respectively. The associations were stronger still for AKIN stage 2–3 with respective ORs 4.00 (CI = 2.10–7.62, p = 1.05 × 10^−5^, FDR = 0.003) and 4.03 (CI = 2.09–7.77, p = 1.88 × 10^−5^, FDR = 0.003) for each minor allele homozygote. We observed no significant association between these SNPs and AKI in the smaller subset of African Americans.

**Conclusion:**

In Caucasian patients with ALI, the presence of minor alleles in two SNPs in *NFKBIA* was strongly associated with the development of AKI.

**Trial registration:**

NCT00281268. Registered 20/01/2006.

**Electronic supplementary material:**

The online version of this article (doi:10.1186/s13054-015-1084-5) contains supplementary material, which is available to authorized users.

## Introduction

Acute kidney injury (AKI) independently predicts increased in-hospital and long-term mortality [1]. The incidence of severe AKI requiring dialysis has increased approximately 10 % per year in the USA between 2000 and 2009 [2], and the mainstay of AKI treatment is still limited to supportive care with or without dialysis [3]. Worldwide, severe AKI is present in approximately 6 % of critically ill patients admitted to intensive care units, predominantly in patients with septic shock (in approximately 48 %), where AKI is associated with mortality of 60 % [4].

A propensity towards inflammation and programmed cell death or apoptosis of tubular cells may be important to the pathophysiology of AKI [5–7]. Fas is a trans-membrane protein in the tumor necrosis factor (TNF) family that, upon binding Fas ligand (FasL), can induce inflammation and apoptosis [8]. *FAS* gene is constitutively expressed by renal tubular epithelial cells at baseline, in balance with cell survival signals [9, 10], and is upregulated in both acute and chronic kidney disease, leading to apoptosis and inflammation [9, 11, 12]. Animal data suggest that Fas is a key mediator of apoptosis and inflammation in AKI [11, 12]. In mice with a loss of function mutation in FasL (referred to as the *gld* mutation), Ko *et al.* observed less AKI after bilateral renal ischemia reperfusion injury (IRI) compared to wild-type mice [13]. They also demonstrated that *gld* mice had fewer TNF-α producing lymphocytes in the kidneys and renal lymph nodes, and that pharmacologic blockade of FasL prevented AKI in wild-type mice after IRI. In patients with acute lung injury (ALI) levels of circulating FasL are associated with changes in serum creatinine [14]. These findings implicate the Fas pathway in the pathophysiology of AKI and as a potential therapeutic target in AKI.

We have previously reported that variants in *FAS* are associated with ALI [15]. Mounting data suggest that there is crosstalk between the kidneys and lungs, and that AKI and ALI are closely interconnected [14, 16–19]. In this study, we hypothesized that variation in genes involved in inflammation and apoptosis from the *FAS* pathway might be associated with development of AKI in patients with ALI. Using data obtained in the Fluid and Catheter Treatment Trial (FACTT), a randomized controlled trial in critically ill patients with ALI [20, 21], we investigated whether genetic variation in single nucleotide polymorphisms (SNPs) in *FAS* and related genes is associated with AKI.

## Materials and methods

### Study population

FACTT was a 2 × 2 factorial randomized trial, conducted by the National Heart, Lung, and Blood Institute (NHLBI) Acute Respiratory Distress Syndrome (ARDS) Clinical Trials Network. FACTT included 1,000 patients with ALI for <48 h, randomized to receive conservative vs. liberal fluid management in one arm, and pulmonary artery catheter (PAC) vs. central venous catheter (CVC) in the other arm; eligibility and exclusion criteria have been previously described [20, 21]. To be eligible for enrollment in FACTT, patients were required to be intubated and on positive-pressure ventilation, have a ratio of the partial pressure of arterial oxygen (PaO2) to the fraction of inspired oxygen (FiO2) <300, and have bilateral infiltrates on chest radiography consistent with pulmonary edema without evidence of left atrial hypertension for <48 h. An additional enrollment requirement was the intent by the primary physician to insert a CVC. Written informed consent was obtained from participants or legally authorized surrogates in the original FACTT study and available to use for secondary analysis. Further details about the consent process can be found in the FACTT study [21]. There was no difference in the incidence of AKI or in receipt of dialysis between the FACTT treatment arms, although these were secondary outcomes of the original studies [21, 22].

### Genotyping

Among the 1,000 FACTT patients, 310 Caucasians and 91 African-Americans provided consent for genetic testing. The University of Washington Institutional Review Board approved this ancillary study. We previously showed that the baseline characteristics of genotyped vs. non-genotyped patients in FACTT are very similar [15]. A priori, we performed our analyses separately by race, to avoid confounding by race. We genotyped 367 SNPs in 45 genes (Additional file 1: Table S1), chosen from an annotated pathway diagram of genes involved in the Fas/FasL pathway [8, 23]. We used re-sequencing information available through the NHLBI Program in Genomic Applications [24], the National Institute of Environmental Health Sciences Environmental Genome Project [25], and the International HapMap Project (Release #19) [26] to calculate linkage disequilibrium (LD) bins (excluding SNPs with minor allele frequency (MAF) <0.05 and *r*^2^ < 0.8) for each of the 45 genes chosen, using LD SELECT on the genome variation server [27–30]. We then selected the final 367 TagSNPs from within the identified LD bins by prioritizing those previously reported to be associated with a disease or quantitative trait and those more likely to have functional significance (non-synonymous > synonymous > untranslated region). The selected TagSNPs covered over 95 % of the common LD bins within the candidate genes. The SNPs and their associated genes are listed in the Additional file 1: Table S2. We used a commercially available Illumina GoldenGate BeadXpress (San Diego, USA) system to genotype the selected SNPs [28, 29].

### Quality control

**Fig. 1:**
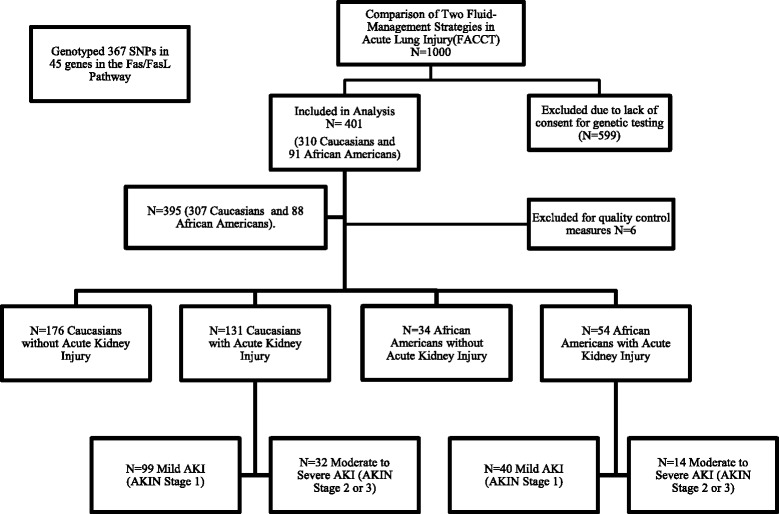
Flow chart of patient allocation during the study. *SNP* single nucleotide polymorphism, *FasL* Fas ligand, *AKIN* Acute Kidney Injury Network

Resulting genotype information underwent quality control at the SNP and subject level. We excluded six patients for whom <80 % of the SNPs were successfully genotyped (mean individual call rate 98.5 %) and we excluded 23 SNPs for which <80 % of patients were successfully genotyped (mean SNP call rate 93.7 %). After stratifying the genotyped patients by self-identified race (Caucasian or African-American), we excluded an additional 30 and 51 SNPs in Caucasians and African-Americans, respectively, for which the MAF was <0.05. We excluded an additional 30 SNPs in Caucasians and 13 in African-Americans, respectively, for which *p* values among controls for Fisher’s exact Hardy Weinberg equilibrium were <1 × 10^−3^ [31]. After these quality control measures, 284 (77.4 %) and 280 (76.3 %) SNPs remained from 307 Caucasian subjects and 88 African-American subjects, respectively. A flow diagram of patient groups is provided in Fig. 1.

### Outcome

Our definition of AKI was adapted from the AKI Network (AKIN) definition [32, 33]. While the AKIN definition utilizes a sequential, incremental increase in serum creatinine (SCr) within 48 h, we used peak compared to trough SCr within the first 2 days following study enrollment. In doing so, we aimed to increase the sensitivity for ALI-associated AKI, which may have occurred before enrollment in FACTT, as patients could have enrolled up to 48 h after the onset of ALI. Stage 1 and higher AKI was defined as an increase in SCr > =0.3 mg/dl or > =50 %. Hourly urine output was not available in the FACTT database.

### Primary analysis

In our primary analysis, we assessed the risk of AKI by genotypes using logistic regression, assuming an additive genetic effects model, i.e., each additional copy of the minor allele (0, 1 or 2) is associated with an incremental change in risk of AKI. We performed the analysis separately for each race (Caucasian and African-American). A priori, we decided to adjust for gender, age, and FACTT treatment arm. We did not adjust for sepsis because we were concerned it may sit within the causal pathway between genotype and AKI. We corrected for multiple comparisons by using a false discovery rate (FDR) threshold <0.1, which estimates that less than 10 % of the associations with an FDR value at or below this level are false positives (29). This is intermediate between using a raw *p* value (least conservative) and Bonferroni correction (most conservative), and accounts for the fact that many of the genotypes are in partial LD and, thus, many of the hypothesis tests are correlated. We used Fisher’s exact test to evaluate genotype frequencies for Hardy-Weinberg equilibrium. Stata 11.2 and Golden Helix SNP & Variation Suite 7 were used for the statistical analyses. Assuming an MAF of 20 % on average, we estimated a power of 80 % to detect an odds ratio (OR) of 1.65 or greater in the Caucasian patients in our study.

### Sensitivity analysis

In sensitivity analysis, we compared the genotypes in those without AKI to those with severe AKI (modified AKIN stage 2 and 3). In this analysis, patients subcategorized as having severe AKI had an increase in SCr >100 %, or had a rise in SCr to 4.0 mg/dl and an increase of at least 0.5 mg/dl. Patients with stage-1 AKI were excluded from the sensitivity analysis, thus excluding patients who may have been misclassified as having AKI with a minimal increase in SCr [34]. Similar to the primary analysis, we assessed the risk of AKI by genotypes using logistic regression with an additive model, and stratified by race. Clinical outcomes including receipt of dialysis, mortality, ventilator-free days, and ICU-free days are outlined by AKI stage in Additional file 1: Tables S3a and b.

## Results

Baseline characteristics for patients in FACTT included in this study are outlined in Table 1 (Caucasians) and Additional file 1: Table S4 (African-Americans). Among the 307 Caucasian patients from FACTT whom we genotyped, 176 (57.3 %) did not develop AKI (AKIN stage 0), while 99 (32.3 %) developed mild AKI (AKIN stage 1), and 32 (10.4 %) developed moderate to severe AKI (AKIN stage 2 or 3) within the first 2 days of study enrollment (Table 1). Among 88 African-Americans, 34 (38.6 %) did not develop AKI, while 40 (45.5 %) developed mild, and 14 (15.9 %) developed moderate to severe AKI stage during the same time period (Additional file 1: Table S4).

**Table 1:** Baseline characteristics of Caucasians in FACTT with genotype data (n = 307)

Characteristic	No AKI^a^	Stage 1 AKI^a^	Stage 2 or 3 AKI^a^
	n = 176 (57.3 %)	n = 99 (32.3 %)	n = 32 (10.4 %)
Age, mean years +/− SD	50.4 +/− 14.6	49.9 +/− 16.3	48.8 +/− 16.1
Male gender	48.9 %	52.5 %	62.5 %
Primary ALI risk factor, n (%)			
Aspiration	30 (17.1 %)	20 (20.2 %)	2 (6.3 %)
Multiple transfusions	2 (1.1 %)	2 (2.0 %)	0
Pneumonia	81 (46.0 %)	46 (46.5 %)	11 (34.4 %)
Sepsis	30 (17.1 %)	21 (21.2 %)	17 (53.1 %)
Trauma	17 (9.7 %)	6 (6.1 %)	1 (3.1 %)
Other	16 (9.1 %)	4 (4.0 %)	1 (3.1 %)
APACHE III score, mean +/− SD^b^	84.4 +/− 25.2	98.8 +/− 30.4	117.4 +/− 26.3
Randomization arm, n (%)			
PAC/fluid liberal	47 (26.7 %)	30 (30.3 %)	11 (34.4 %)
PAC/Fluid	43 (24.4 %)	17 (17.2 %)	11 (34.4 %)
Conservative CVC/fluid	39 (22.2 %)	26 (26.3 %)	8 (25.0 %)
Liberal CVC/fluid	47 (26.7 %)	26 (26.3 %)	2 (6.3 %)
Vasopressor use^c^	55 (31.3 %)	35 (35.4 %)	19 (59.4 %)
Patient location	86 (48.9 %)	58 (58.6 %)	19 (59.4 %)
MICU SICU	21 (11.9 %)	9 (9.1 %)	0
MICU/SICU	41 (23.3 %)	23 (23.2 %)	4 (12.5 %)
Trauma	16 (9.1 %)	6 (6.1 %)	6 (18.8 %)
Other	12 (6.8 %)	3 (3.0 %)	
Baseline creatinine^d^, mg/dl (SD)	0.9 (0.4)	1.4 (0.6)	2.1 (1.2)

^a^Acute kidney injury (*AKI*) study definitions: no AKI = <0.3 mg/dl and <50 % increase in serum creatinine (SCr); stage 1 AKI = > =0.3 mg/dl and > = 50 % increase in SCr; stage 2 or 3 AKI = >100 % increase in SCr. ^b^Missing acute physiology and chronic health evaluation (*APACHE*) score for 9 patients, 5 five patients, and patients with no AKI, stage 1 and 2 or 3 AKI, respectively. ^c^Vasopressor use in the 24 h before Fluid and Catheter Treatment Trial (FACTT) enrollment. ^d^Baseline creatinine based on average SCr in the 24 h before enrollment in FACTT. *ALI* acute lung injury, *PAC* pulmonary artery catheter, *CVC* central venous catheter, *MICU* medical intensive care unit, *SICU* surgical intensive care unit

### Primary analysis

**Fig. 2:**
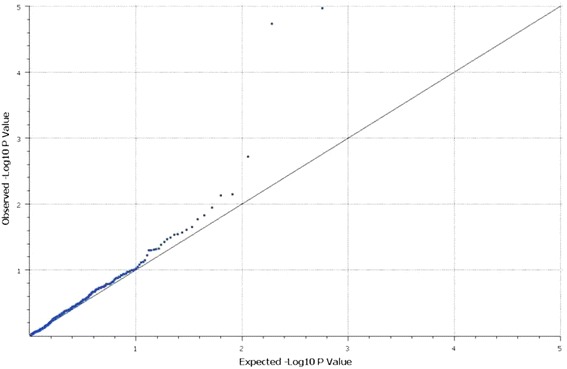
Observed versus expected associations between Fas pathway single nucleotide polymorphisms (SNPs) and risk of acute kidney injury (AKI) (stage 1+ vs. stage 0) in the Fluid and Catheter Treatment Trial (FACTT) for Caucasians

The association tests from the primary analysis are graphically depicted in a quantile-quantile (Q-Q) plot in Fig. 2 for Caucasians and Additional file 2: Figure S1 for African-Americans. The results from association tests for all the SNPs tested are outlined in Additional file 1: Tables S5 and S6 for Caucasians and African-Americans, respectively.

In Caucasians, we observed 22 nominal (*p* <0.05) associations with AKI (Table 2) and two of these associations remained significant after adjustment for multiple hypothesis testing (FDR <0.1): rs1050851 and rs2233417 (Tables 2 and 3). Both of these SNPs are found in *NFKBIA*; rs1050851 is a synonymous coding SNP in exon 2 and rs2233417 is located within intron 4 (based on dbSNP) [35]. For each of these SNPs, the minor allele was associated with increased risk for AKI. For rs1050851, each copy of the A allele was associated with greater than two-fold increase in risk for AKI (OR 2.34, 95 % CI = 1.58–3.46, *p* = 1.06 × 10^−5^, and FDR = 3.00 × 10^−3^). For rs2233417, each copy of the A allele was associated with greater than two-fold increase in the risk for AKI (OR 2.46, CI = 1.61–3.76, *p* = 1.81 × 10^−5^, and FDR = 2.57 × 10^−3^).

**Table 2:** Fas/Fas ligand pathway polymorphisms associated (*p* <0.05) with acute kidney injury (AKI) susceptibility in Caucasian subjects from the Fluid and Catheter Treatment Trial

Gene	SNP	OR (95 % CI)^a^	*P* value	FDR-adjusted *P* value	MAF
*NFKBIA*	rs1050851	2.34 (1.58–3.46)	1.06 × 10^−5^	<0.003	0.18
*NFKBIA*	rs2233417	2.46 (1.61–3.76)	1.81 × 10^−5^	<0.003	0.15
*CFLAR*	rs12105811	0.51 (0.33–0.79)	0.002	0.18	0.25
*NFKBIA*	rs7157810	1.70 (1.15–2.51)	0.007	0.50	0.18
*FASLG*	rs5030772	1.80 (1.16–2.79)	0.007	0.41	0.11
*BCL2L1*	rs1484994	0.63 (0.44–0.91)	0.011	0.54	0.35
*BCL2L1*	rs6060621	0.65 (0.45–0.92)	0.015	0.60	0.35
*TIRAP*	rs1786697	0.60 (0.39–0.92)	0.017	0.60	0.23
*RELA*	rs10896027	1.49 (1.06–2.11)	0.022	0.69	0.34
*MAP3K1*	rs33330	0.64 (0.43–0.95)	0.024	0.69	0.34
*NFKBIA*	rs8904	0.67 (0.47–0.96)	0.027	0.69	0.41
*CFLAR*	rs4482462	0.65 (0.44–0.96)	0.028	0.67	0.27
*NFKBIA*	rs696	0.68 (0.48–0.96)	0.029	0.63	0.41
*DAXX*	rs2239839	1.50 (1.03–2.18)	0.032	0.65	0.26
*CFLAR*	rs6728771	0.66 (0.45–0.97)	0.034	0.64	0.27
*MAP3K5*	rs911179	0.70 (0.50–0.98)	0.037	0.66	0.48
*CASP9*	rs1862710	0.71 (0.51–0.99)	0.041	0.68	0.49
*MYD88*	rs6853	0.61 (0.38–1.00)	0.047	0.76	0.16
*BCL2L1*	rs6058381	0.53 (0.28–1.02)	0.048	0.72	0.09
*TLR6*	rs3821985	0.71 (0.50–1.00)	0.049	0.70	0.38
*CFLAR*	rs7583529	0.68 (0.46–1.00)	0.0497	0.67	0.26
*MAP3K5*	rs1570054	0.71 (0.50–1.00)	0.0498	0.64	0.40

^a^Risk of AKI (stage 1 or above) with each additional copy of the minor allele. *SNP* single nucleotide polymorphism, *OR* odds ratio, *FDR* false discovery rate, *MAF* minor allele frequency

**Table 3:** Genotype counts for rs1050851 and rs2233417 by acute kidney injury (AKI) stage in Caucasian subjects from the Fluid and Catheter Treatment Trial

AKIN Stage	rs1050851 Genotype counts (% per AKI stage) (n = 305^a^)				rs2233417 Genotype counts (% per AKI stage) (n = 305^b^)			
	AA	AG	GG	Total	AA	AG	GG	Total
0	5 (2.9 %)	53 (30.5 %)	116 (66.7 %)	174	3 (1.7 %)	45 (25.7 %)	127 (72.6 %)	175
1	9 (9.1 %)	42 (42.4 %)	48 (48.5 %)	99	4 (4.1 %)	41 (41.8 %)	53 (54.1 %)	98
2–3	6 (18.8 %)	17 (53.1 %)	9 (28.1 %)	32	5 (15.6 %)	15 (46.9 %)	12 (37.5 %)	32
Total	20	112	173	305	12	101	192	305

^a^Two patients had missing genotype for rs1050851; neither had AKI. ^b^Two patients had missing genotype for rs2233417; one had stage-1 AKI and the other did not have AKI (stage 0). *AKIN* Acute Kidney Injury Network

In African-American patients, we observed 13 nominal (*p* <0.05) associations with AKI, however none remained significant after adjustment for multiple hypothesis testing (Additional file 1: Tables S6a and S7). There were no significant associations observed between rs1050851 (OR 1.92, CI = 0.51–7.27, p = 0.87, FDR = 0.93) or rs2233417 (OR 0.90, CI = 0.23–3.52, *p* = 1.00, FDR = 1.00) and AKI. Given that there were only 88 African-Americans available for genotyping, our study did not have adequate power to exclude the presence of an association (Additional file 1: Table S6a). We also tested recessive genetic models for associations between these two SNPs and AKI and there were no statistically significant results in either population. Thus, we used an additive genetic model for the remainder of the analyses.

### Sensitivity analysis

The associations for rs1050851 and rs2233417 with AKI were stronger when we limited the analyses to patients with moderate to severe AKI or no AKI (OR 4.00, CI = 2.10–7.62, *p* = 2.78 × 10^−5^, and FDR = 2.97 × 10^−3^ for rs1050851 and OR 4.03, CI = 2.09–7.77, *p* = 4.68 × 10^−5^, and FDR = 2.66 × 10^−3^ for rs2233417). We observed no association between rs1050851 (OR 3.76, CI = 0.79–17.77, *p* = 0.39, and FDR = 1.00) or rs2233417 (OR 1.13, CI = 0.18–7.32, *p* = 0.82, FDR = 1.00) and risk of moderate-severe AKI in African-Americans. Using an FDR threshold <0.1, no other associations were significant in our sensitivity analysis in either Caucasians (Tables 2 and 4) or African-Americans (Additional file 1: Tables S7a and b) although we did observe nominal (*p* <0.05) associations with 15 SNPs and 10 SNPs in Caucasians and African-Americans, respectively (Table 4 and Additional file 1: Table S6b, respectively).

**Table 4:** Top 15 Fas/Fas ligand pathway polymorphisms in the Fluid and Catheter Treatment Trial associated with acute kidney injury (AKI) (stage 2–3 vs. stage 0) in Caucasians (stage 1 excluded)

Gene	SNP	OR (95 % CI)^a^	*P* value	FDR	MAF
*NFKBIA*	rs1050851	4.00(2.10–7.62)	1.05 × 10^−5^	<0.003	0.18
*NFKBIA*	rs2233417	4.03(2.09–7.77)	1.88 × 10^−5^	<0.003	0.15
*BAX*	rs4645878	0.12 (0.02–0.90)	0.004	0.41	0.12
*TIRAP*	rs3802814	2.45 (1.28–4.72)	0.008	0.55	0.14
*BCL2L1*	rs6058381	0.13 (0.02–1.02)	0.008	0.48	0.09
*BAX* ^*b*^	rs2387583	0.20 (0.05–0.89)	0.009	0.42	0.13
*NFKBIA*	rs7157810	2.19 (1.22–3.94)	0.009	0.38	0.18
*BAX*	rs1010103	0.21 (0.05–0.92)	0.01	0.38	0.13
*LY96*	rs16938758	2.22 (1.21–4.09)	0.01	0.34	0.17
*ALS2CR1*	rs17468277	2.36 (1.15–4.88)	0.02	0.67	0.10
*DAXX*	rs2239839	2.05 (1.10–3.82)	0.02	0.61	0.26
*CASP8*	rs1045485	2.32 (1.12–4.78)	0.03	0.64	0.11
*CASP9*	rs1820204	1.84 (1.04–3.27)	0.03	0.770	0.47
*BCL2*	rs1542578	0.55 (0.31–0.97)	0.04	0.72	0.49
*TIRAP*	rs8177343	1.98 (1.03–3.79)	0.04	0.79	0.16

^a^Risk of AKI (stage 1 or above) with each additional copy of the minor allele. ^b^Also nominally associated with stage 2–3 AKI in African-Americans (*p* <0.05). *SNP* single nucleotide polymorphism, *OR* odds ratio, *FDR* false discovery rate, *MAF* minor allele frequency

## Discussion

We now report the first evidence that genetic variation in *NFKBIA* is associated with susceptibility to AKI, discovered in this genetic association study examining the risk of AKI related to Fas and related genes. Fas ligation leads to a series of intracellular signaling events, culminating in activation of the death-inducing signaling complexes (DISCs), which promote the activation of caspase-8-mediated apoptosis. Notably, there are several important negative regulators of Fas-induced apoptosis including B-cell lymphoma-2 (*BCL2*), FLICE-like inhibitory protein (*FLIP*), and baculoviral IAP repeat-containing protein 2 (*IAP1*), all three of which demonstrate NF-kB-dependent transcription [36, 37]. *NFKBIA* codes for the cytoplasmic protein, I-Kappa-B-Alpha (IKBA), that inhibits NF-κB-mediated transcriptional responses by binding to NF-κB and retaining it in the cytoplasm [38].

Genetic variation in the promoter region in *NFKBIA* is associated with differential susceptibility to pediatric lung disease [39]. NF-κB is a transcription factor that regulates activation of the immune and inflammatory responses [38], has been shown to regulate the expression of FasL [40], and is believed to be a mediator of renal injury in obstructive AKI, [41] and it participates in the renal development pathway [42]. Taken together, these findings suggest that *NFKBIA* might play an important pathophysiologic role in AKI [38, 40–44].

Our study adds to mounting evidence supporting a role for genetic variation in modulating risk of AKI. Prior work has identified associations between genetic variation in inflammatory pathways and AKI [45]. For example, Jaber et al. found SNPs in the promoter region of *TNFA* and *IL10* were associated with decreased risk of death in patients with AKI, who required dialysis [46]. An SNP in the *IL6* has been associated with AKI although follow up studies have not been able to replicate this association [47–49]. Our study focused on genes implicated directly or indirectly with the Fas/FasL and apoptotic pathways so we did not genotype variation in these genes.

Polymorphisms in *BCL2*, rs8094315 and rs12457893, were recently reported to be associated with decreased risk of developing AKI in patients with septic shock [5]. Our study included rs8094315 as well as other SNPs in *BCL2* but did not include rs12457893. We did not find an association between SNPs in *BCL2* and susceptibility to AKI but our study subjects differed significantly from the prior study. Our study included a heterogeneous group of patients who were enrolled for an interventional trial in acute respiratory distress syndrome (ARDS) while the prior study included patients with septic shock, some of whom went on to develop ARDS. The difference in underlying illness leading to ICU admission and subsequent organ dysfunction may explain the differences in our findings. No prior studies have examined variation in *NFKBIA* in relation to risk for AKI. Future studies in large critically ill populations with well-defined AKI and associated clinical risks will be necessary to clarify the role of genetic variation in determining risk of AKI.

Our study has several limitations. First, not all patients in the FACTT consented to genetic testing and so we only have genotypic data for a subset of the original 1,000 patients, however, FACTT patients who were genotyped were no different to those who were not genotyped [15]. Second, multiple hypothesis testing will increase the likelihood of type I (false positive) errors. However, we mitigated this possibility by using an FDR cutoff. Third, our results in Caucasian subjects may not be generalizable to all populations. Indeed, while the sample size was very limited, our findings among African-Americans did not replicate the associations between AKI risk and *NFKBIA*. Future studies will need to focus on much larger populations of non-Caucasians to address this issue. Fourth, our definition of AKI differs from prior studies reporting the incidence of AKI in the FACTT. We used a more inclusive AKI definition in order to minimize the number of AKI cases misclassified as controls and maximize statistical power for our genetic study, and this resulted in a higher incidence (42 %?) than reported by Liu et al. (30.6 %) [22]. Additional studies in prospectively enrolled subjects with known pre-hospital renal function will be necessary to clarify which definition better represents true AKI. Finally, while *NFKBIA* is certainly biologically plausible as a potential contributor to AKI pathophysiology, further work will be needed to clarify whether the variants we report as associated with AKI risk have any functional effect on expression or function of *NFKBIA*.

Our study has significant strengths. There is a strong biologic rationale for the hypothesis that the Fas pathway is important in AKI, as outlined above. The high-quality clinical phenotypes and covariates available through the FACTT provided us with an exceptional opportunity to link genotype data with AKI with maximal precision. Finally, although our findings will need to be replicated in independent populations, our study is the largest to evaluate genetic risks for AKI in critically ill patients from a mixed medical and surgical ICU population.

## Conclusions

This is the first human study to describe an association between polymorphisms in *NFKBIA* and AKI. Genotypes in two SNPs in *NFKBIA* were associated with increased AKI occurrence in Caucasians with ALI: rs1050851 (coding-synonymous) and rs2233417 (intronic). This study identifies a potential role for *NFKBIA* in the pathophysiology of AKI in the critically ill.

## Key messages

- This is the first human study to describe associations between nuclear factor of kappa light polypeptide gene enhancer in B-cells inhibitor, alpha (*NFKBIA*) and acute kidney injury in patients with acute respiratory distress syndrome
- Two single nucleotide polymorphisms rs1050851 and rs2233417 are both associated with acute kidney injury
- The OR for rs1050851 was 2.34 (95 % CI = 1.58–3.46, *p* = 1.06 × 10^−5^, FDR = 0.003) for acute kidney injury and for rs2233417 it was 2.46 (95 % CI = 1.61–3.76, *p* = 1.81 × 10^−5^, FDR = 0.003) for each minor allele, respectively
- The associations were stronger still for AKIN stage 2–3 with respective ORs of 4.00 (95 % CI = 2.10–7.62, *p* = 1.05 × 10^−5^, FDR = 0.003) and 4.03 (95 % CI = 2.09–7.77, *p* = 1.88 × 10^−5^, FDR = 0.003) for each minor allele homozygote

## Additional files

Additional file 1: Table S1. Genes (n = 45) in the Fas/Fas ligand (FasL) pathway characterized using tag single nucleotide polymorphisms (tagSNPs). **Table S2.** TagSNPs genotyped using Illumina GoldenGate genotyping assays. **Table S3a.** Outcomes by acute kidney injury (AKI) stage in Caucasians. **Table S3b.** Outcomes by AKI stage in African-Americans. **Table S4.** Characteristics of African-Americans in the Fluid and Catheter Treatment Trial (FACTT) with genotype data (n = 88). **Table S5a.** Odds ratios for AKI associated with individual SNPs among Caucasians (comparing no AKI vs. stage 1–3 AKI). **Table S5b.** Odds ratios for AKI associated with individual SNPs among Caucasians (comparing no AKI vs. stage 2–3 AKI). **Table S6a.** Odds ratios for AKI associated with individual SNPs among African-Americans (comparing no AKI vs. stage 1–3 AKI). **Table S6b.** Odds ratios for AKI associated with individual SNPs among African-Americans (comparing no AKI vs. stage 2–3 AKI). **Table S7a.** Fas/FasL pathway polymorphisms in the FACTT associated (*p* <0.05) with AKI susceptibility in African-American subjects from the FACTT. **Table S7b.** Fas/FasL pathway polymorphisms in the FACTT associated (*p* <0.05) with AKI (stage 2–3 vs. stage 0) in African-Americans (Stage 1 excluded). (XML 972 bytes) Additional file 2: Figure S1. Observed versus expected associations between Fas pathway single nucleotide polymorphisms (SNPs) and risk of acute kidney injury (AKI) (stage 1+ vs. stage 0) in African-Americans from the Fluid and Catheter Treatment Trial (FACTT). (DOC 152 kb)

## Abbreviations

AKI: acute kidney injury
AKIN: Acute Kidney Injury Network
ALI: acute lung injury
ARDS: acute respiratory distress syndrome
CVC: central venous catheter
FACTT: Fluid and Catheter Treatment Trial
FasL: Fas ligand
FDR: false discovery rate
IRI: ischemia reperfusion injury
LD: linkage disequilibrium
MAF: minor allele frequency
MPO: myeloperoxidase
NFKBIA: nuclear factor of kappa light polypeptide gene enhancer in B-cells inhibitor, alpha
OR: odds ratio
PAC: pulmonary artery catheter
Q-Q: quantile-quantile
SCr: serum creatinine
SNP: single nucleotide polymorphism
TNF: tumor necrosis factor
