# Supplementary material for: Associations between single nucleotide polymorphisms in the FAS pathway and acute kidney injury
Source: Crit Care. 2015 Oct 19;19:368. doi: 10.1186/s13054-015-1084-5 (PMC4610046; doi:10.1186/s13054-015-1084-5)

Appendix - Figure 1. Observed versus expected associations between Fas pathway SNPs and risk for AKI (Stage 1+ vs. Stage 0) in FACTT for African-Americans.


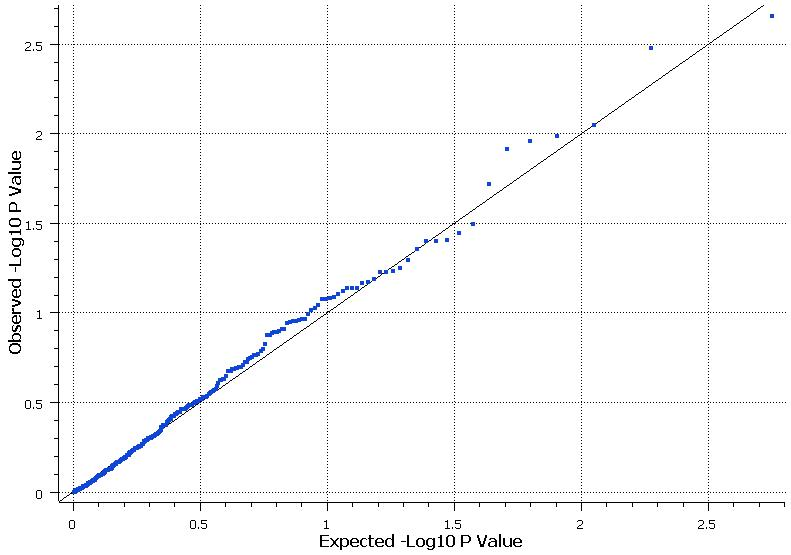

Supplement: Additional file 2: Figure S1. — Observed versus expected associations between Fas pathway single nucleotide polymorphisms (SNPs) and risk of acute kidney injury (AKI) (stage 1+ vs. stage 0) in African-Americans from the Fluid and Catheter Treatment Trial (FACTT). (DOC 152 kb) [file 13054_2015_1084_MOESM2_ESM.doc]
